# P-723. Incidence, Etiology, and Outcomes of Viral Pneumonia in Adults with Cancer at a Tertiary Care Centre in Eastern India: A One Year Prospective Cohort Study

**DOI:** 10.1093/ofid/ofae631.919

**Published:** 2025-01-29

**Authors:** Simran Malik, Sanjay Bhattacharya, Sangeeta Das Bhattacharya

**Affiliations:** Indian Institute of Technology Kharagpur, Kharagpur, West Bengal, India; Tata medical center kolkata, Kolkata, West Bengal, India; Indian Institute of Technology Kharagpur, Kharagpur, West Bengal, India

## Abstract

**Background:**

We identified pneumonia in adult cancer patients admitted to the ICU over one year, delineated viral pneumonia, assessed etiology, and studied clinical outcomes.

**Figure 1:**
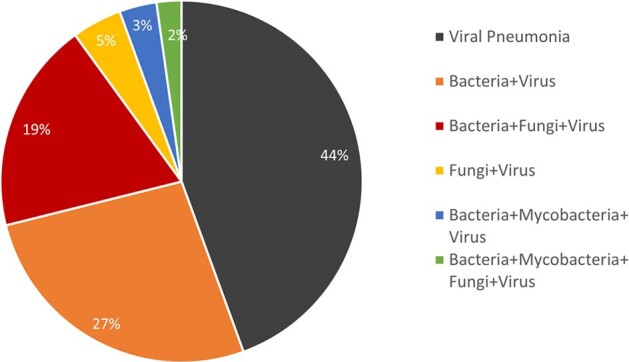
Etiology of pneumonia cases where viruses were detected (n=90)

**Methods:**

Pneumonia cases were clinically and radiologically identified from October 2022-September 2023. Pneumonia was categorized as community-acquired or healthcare-associated. Viral detection was done using Qiagen’s QIAstat-Dx Respiratory SARS-CoV-2 Panel and bioMeriéux’s BioFire® FilmArray® Pneumonia Panel. Bacterial, fungal, and mycobacterial detection involved microscopy, culture, ELISA, PCR, and CBNAATs.

All cases where bacteria, mycobacteria, and fungi were detected along with viruses were assigned “co-infection/ superadded infections". Frequency of pathogens were compared using a modified Z-test. Seasonality was assessed using the chi-square test for homogeneity. Lengths of hospital and ICU stay were compared using linear regression and mortality was compared using logistic regression and Kaplan-Meier survival analysis.

**Figure 2:**
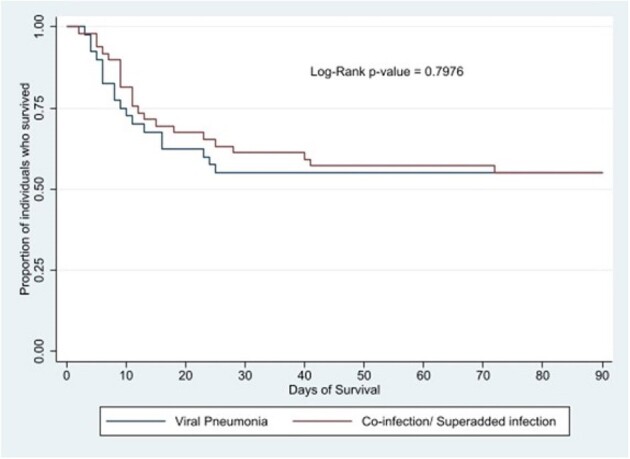
Kaplan-Meier survival analysis for pneumonia cases where viruses were detected stratified by etiology

**Results:**

355 cases of pneumonia were identified. Viruses were detected in 90 (25.4%). Of these 40 were viral pneumonia [incidence: 1.8% (95% CI: 1.3-2.4%)] among 2279 in-patients, and 50 were viral co-infections/ superadded infections (figure 1).

Rhinovirus/ enterovirus (27) was the commonest followed by SARS-CoV2 (15), influenza A (12), and RSV A/B (10). RSV A/B was more common in CAP cases (p=0.005). CMV was more common in HAP (p=0.042). Seasonal variation was seen in influenza A/B only with the highest frequency during monsoon (p=0.000).

*Aspergillus* (23) was most frequently detected alongside viruses, followed by *Klebsiella pneumoniae* (17), *Staphylococcus aureus*, *Pseudomonas aeruginosa*, and *Acinetobacter* (10 each).

The overall length of hospital stay in this cohort was 16.3 days, length of ICU stay was 9.6 days, and 90-day mortality was 54.4%. Length of ICU stay was higher in viral co-infections/ superadded infections (table 2, figure 2).

**Figure:**
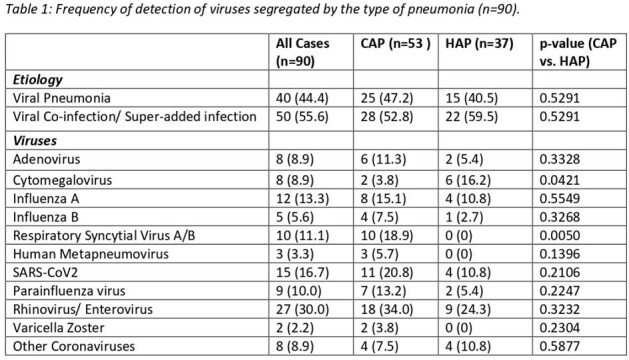

**Conclusion:**

Majority of pneumonia cases with viral detection are co-infections/ superadded infections associated with prolonged ICU stay. There is a need for diagnostic stewardship to ensure rapid diagnosis, and appropriate antimicrobial prophylaxis for antibacterial and antifungal cover.

**Figure:**
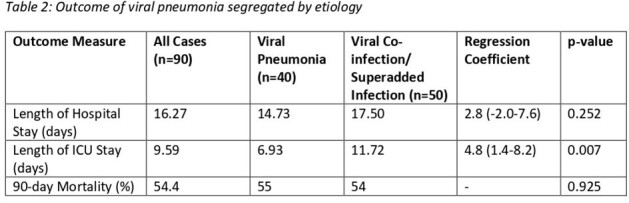

**Disclosures:**

**All Authors**: No reported disclosures

